# Case Report: Area of focus in a case of giant aortic arch pseudoaneurysm following fish bone penetration

**DOI:** 10.3389/fcvm.2025.1633808

**Published:** 2025-10-23

**Authors:** Jianxian Xiong, Jiaxin Cao, Junjian Yu, Peijun Li, Zitao Zeng, Xudong Pan

**Affiliations:** ^1^ Department of Cardiovascular Surgery, First Affiliated Hospital of Gannan Medical University, Ganzhou, China; ^2^ The First Clinical Medical College of Gannan Medical University, Ganzhou, China; ^3^ Department of Cardiovascular Surgery, Beijing Anzhen Hospital, Capital Medical University, Beijing Institute of Heart Lung and Blood Vessel Diseases, Beijing, China

**Keywords:** aortic arch pseudoaneurysm, esophageal fistula, foreign body, emergency surgery, one-stage recovery

## Abstract

**Background:**

Esophageal foreign body impaction represents a common clinical emergency. In severe cases, it can lead to esophageal perforation and aortic injury, potentially causing life-threatening aorto-esophageal fistulas. While immediate intervention is critical, concurrent infection and retained foreign material substantially complicate management.

**Case Presentation:**

We report a rare case of a giant aortic arch pseudoaneurysm secondary to a migratory fish bone. Contrast-enhanced computed tomography (CT) confirmed the diagnosis, prompting urgent surgery. Median sternotomy under cardiopulmonary bypass enabled simultaneous foreign body extraction and aortic perforation repair using autologous pericardium. We reconstructed the aortic arch using a branched stent-graft system via the frozen elephant trunk (FET) technique. Postoperatively, systematic mediastinal irrigation, proactive gastrointestinal decompression, and protocolized enteral nutritional support facilitated complete recovery without requiring secondary esophageal reconstruction.

**Conclusion:**

Aortic injuries resulting from esophageal foreign bodies frequently necessitate a multidisciplinary approach. This case demonstrates the feasibility of a single-stage surgical strategy combining aortic repair via the frozen elephant trunk technique with conservative, non-operative management of the concomitant esophageal injury, facilitated by rigorous postoperative care. Treatment strategies must be individualized based on a comprehensive assessment of the aortic involvement pattern, anatomical location, and coexisting pathological conditions.

## Introduction

Osseous foreign bodies, particularly fish or chicken bones, are a leading cause of esophageal injury and constitute a significant clinical burden. In severe cases (1%–3%), complications such as perforation, retained foreign bodies, mediastinitis, pleural empyema, or life-threatening hemorrhage necessitate surgical intervention (1). Critical risk arises when sharp objects migrate within the mediastinum, potentially injuring the thoracic aorta and precipitating catastrophic hemorrhage or pseudoaneurysm formation—scenarios demanding urgent surgical management. While endovascular therapies may offer temporizing control of bleeding or rupture risk, definitive management typically requires a two-stage approach: initial foreign body extraction with debridement of infected tissue, followed by esophageal repair or reconstruction for persistent fistulae.

Here, we report a notable case of a giant aortic arch pseudoaneurysm secondary to a fish bone, successfully managed through one-stage surgical repair resulting in full recovery without secondary interventions.

## Case description

**Figure 1:**
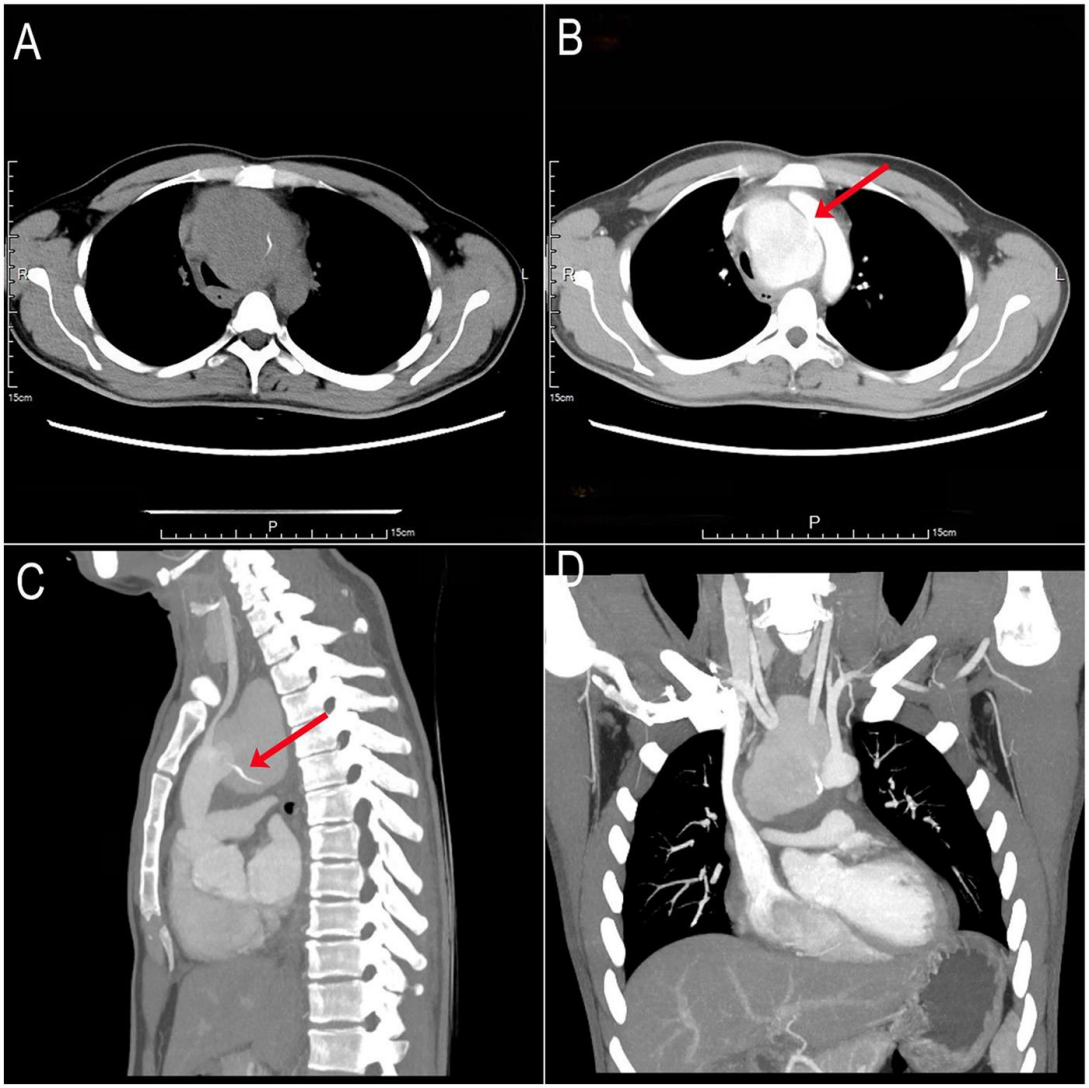
Preoperative non-contrast and contrast-enhanced CT scans. **(A)** Non-contrast CT: Giant mediastinal mass containing a hyperdense foreign body. **(B)** Contrast-enhanced CT: Giant aortic arch pseudoaneurysm (posterior wall) with a 1-cm filling defect (red arrow). **(C,D)** Contrast-enhanced CT: Foreign body tip penetrating toward the aortic arch (red arrow).

A 32-year-old male presented with cough and chest pain exacerbated by swallowing. Twenty days prior, he had accidentally ingested a fish bone but deferred medical evaluation. He subsequently developed high fever, worsening chest pain, and severe coughing. The patient had no significant past medical history and no family history of similar disorders or premature cardiovascular disease. On auscultation, lung fields were clear bilaterally, without rales or rhonchi. Heart sounds were regular, with no pathological murmurs detected. Non-contrast computed tomography (CT) revealed a large mediastinal mass containing a high-density foreign body (Figure 1A). Contrast-enhanced CT demonstrated a giant aortic arch pseudoaneurysm causing significant tracheoesophageal compression and mediastinal shift to the right, along with the retained foreign body (Figure 1B) and a substantial filling defect in the posterior aortic arch wall (Figures 1C,D).

**Figure 2:**
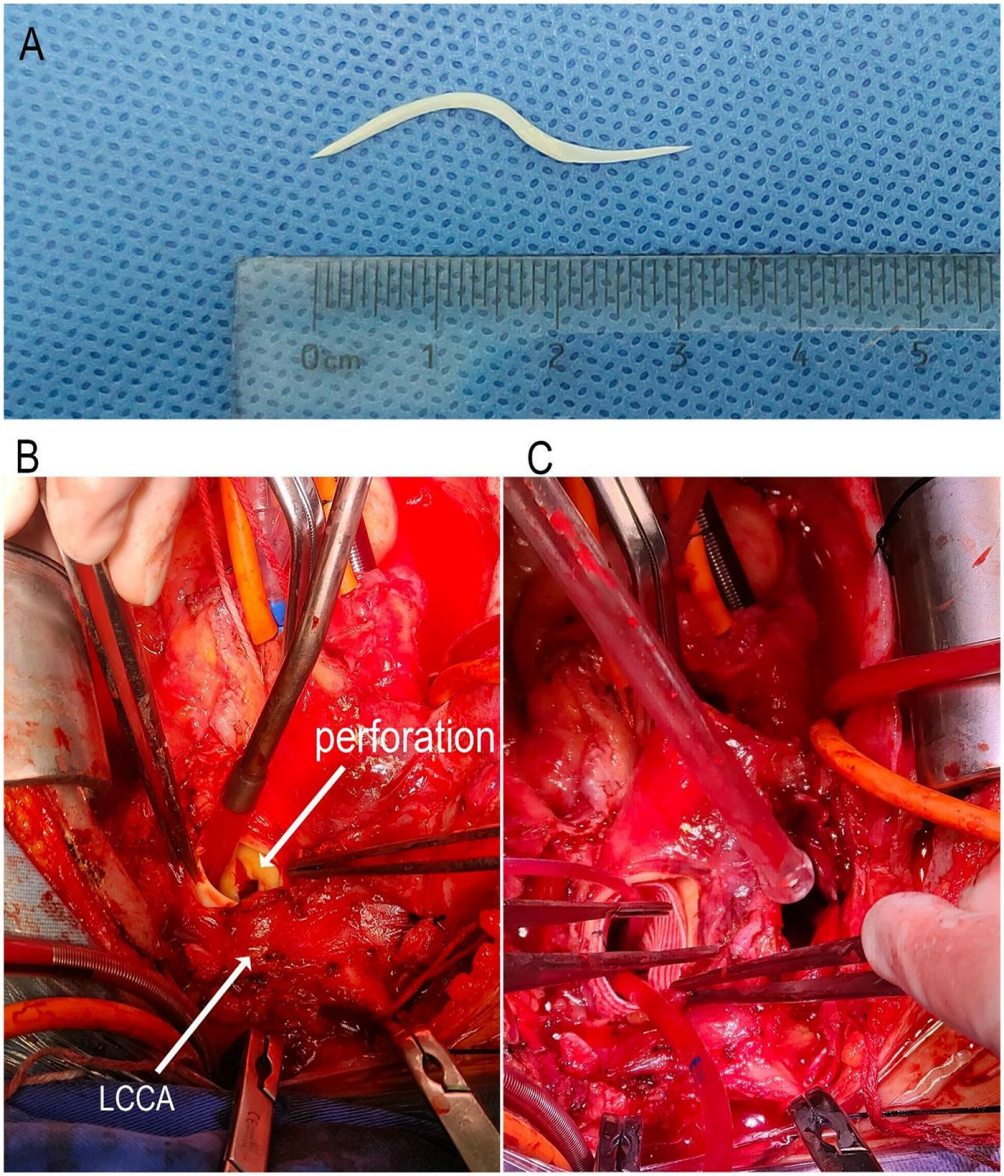
Intraoperative and postoperative imaging. **(A)** Extracted 3-cm S-shaped sharp fish bone. **(B)** Intraoperative view: A cannulation was performed in the LCCA, and bilateral antegrade cerebral perfusion was administered during circulatory arrest. The aortic arch was longitudinally incised, revealing a 1-cm perforation on the posterior wall of the arch, adjacent to the orifice of the LCCA. A sharp fish bone was found penetrating through the perforation site. **(C)** The fish bone was removed, and the perforation was repaired using autologous pericardium. A branched surgical stent graft system was then deployed into the aortic arch and distal segment to ensure coverage of the perforation site. Additionally, the sac of a pseudoaneurysm was opened on the right side of the aortic arch.

Emergency surgery was performed via median sternotomy. Cardiopulmonary bypass (CPB) was established via right axillary artery and right atrial cannulation. Loose fibrinous adhesions were present in the pericardial cavity, with dense adhesions surrounding the aortic arch. Following ascending aortic cross-clamping and cardioplegia administration, circulatory arrest was initiated at 28°C. Bilateral cerebral perfusion was maintained via the right axillary artery and a cannula placed in the left common carotid artery (LCCA). Upon opening the aortic arch, a 1-cm diameter perforation was identified in the posterior wall, containing a sharp fish bone (Figures 2A,B). After removing the foreign body, we repaired the perforation using an autologous pericardial patch. A branched surgical stent graft system (Figure 2C) was deployed using the frozen elephant trunk (FET) technique, with the branch selectively deployed into the left subclavian artery (LSA). The perforation was directly adjacent to the LCCA origin. As the stent graft deployment covered the LCCA ostium, we performed an 8-mm graft bypass from the ascending aorta to the LCCA.

**Figure 3:**
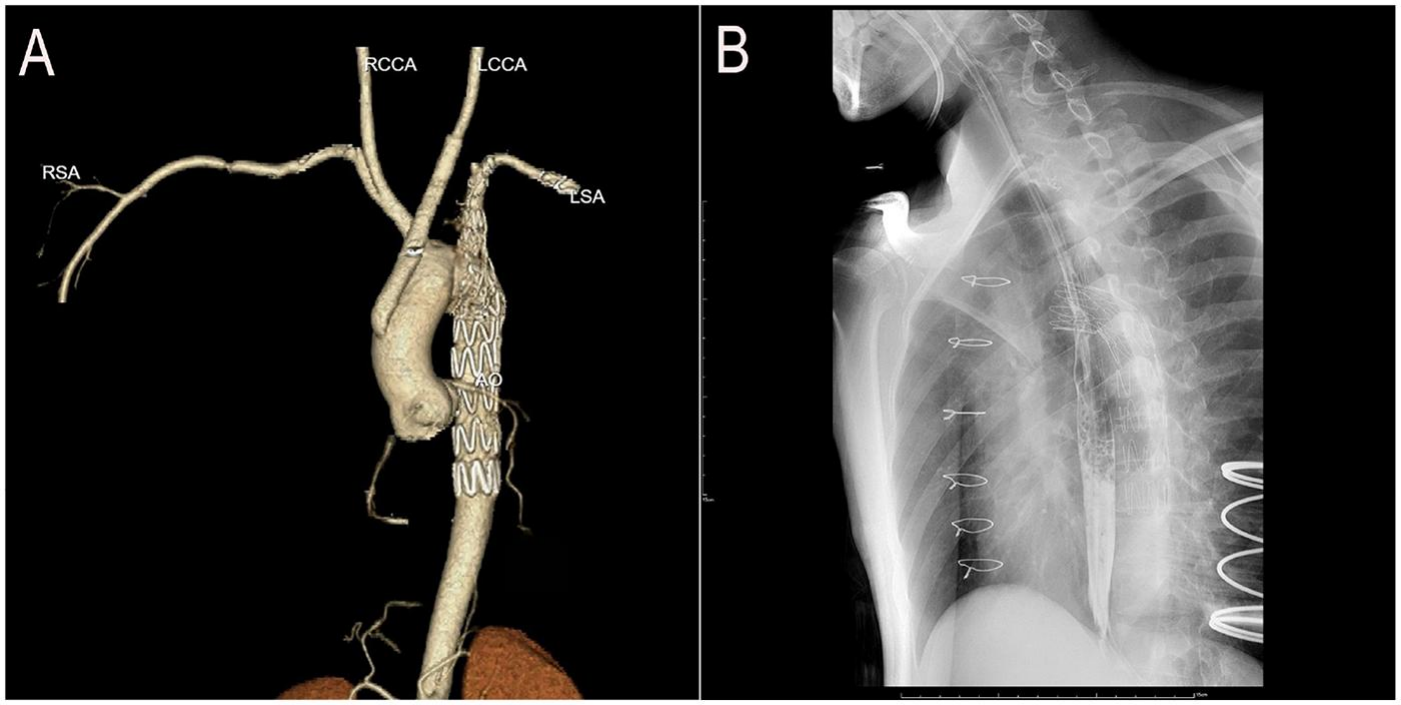
Postoperative imaging follow-up. **(A)** CTA: Restored aortic arch anatomy after stent graft deployment. **(B)** Esophagography: Healed esophageal mucosa with smooth contour and no evidence of contrast extravasation or fistula formation.

**Figure 4:**
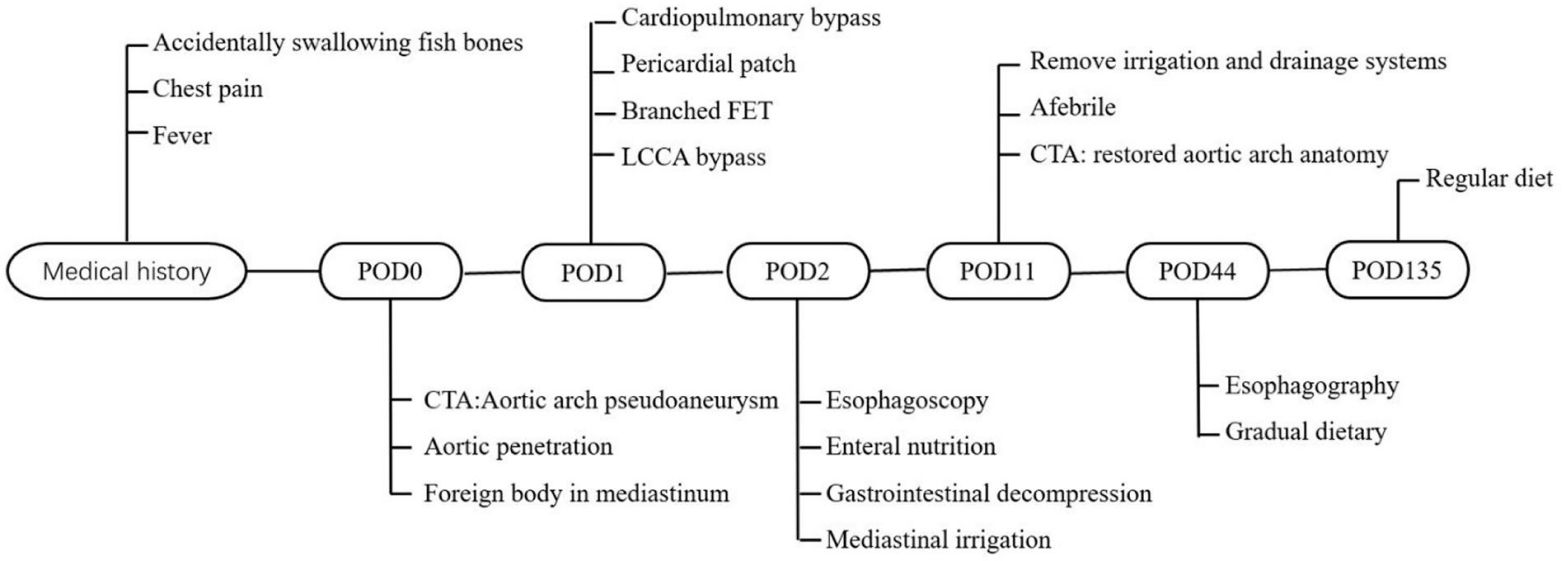
Timeline of the case.

The patient regained consciousness 3 h postoperatively. Esophagoscopy disclosed a 1-cm longitudinal mucosal tear 25 cm from the incisors (Supplementary Video S1), prompting placement of a jejunal feeding tube and gastrointestinal decompression. Mechanical ventilation was discontinued on postoperative day 3. We instituted a regimen of low-volume mediastinal irrigation with normal saline (500 ml every 12 h) combined with low negative pressure drainage, continued for seven days. Following irrigation cessation, the patient was monitored for an additional 48 h. Drainage fluid remained clear and serosanguinous, body temperature normalized, and inflammatory markers (procalcitonin [PCT] and white blood cell count [WBC]) returned to baseline. Bacterial culture of the drainage fluid was negative. Post-cessation computed tomography angiography (CTA) confirmed restored aortic arch anatomy (Figure 3A), allowing removal of the irrigation and drainage systems. Enteral nutrition was maintained for 6 weeks. Subsequent esophagography showed no evidence of esophageal fistula (Figure 3B), enabling gradual dietary advancement. The patient recovered without complications and was discharged (Figure 4). At the three-month postoperative follow-up, he remained asymptomatic but declined follow-up gastroscopy and aortic CTA.

## Patient perspective

(Informed consent was obtained from the patient for publication of this perspective.)

The patient initially dismissed the swallowed fish bone as a minor issue and experienced only mild discomfort for several days. This false sense of security was shattered ten days later, when he developed severe, sharp retrosternal pain during a meal—a sensation he described as “being stabbed, leaving no doubt something was critically wrong.” He sought emergency care immediately. Upon diagnosis, he expressed profound shock and fear: “I couldn't believe a small bone could threaten my life.” However, the surgical team's clear communication helped him understand the urgency and risks, and he actively participated in the decision to proceed with surgery, placing full trust in the medical team.

The postoperative recovery proved challenging, marked by pain and a prolonged hospital stay. He credited his family's support and the medical team's responsiveness as crucial to his emotional coping. “The doctors listened patiently to all my concerns,” he noted, highlighting this as the most valuable aspect of his care. Currently, he has resumed a normal diet and all daily activities and feels “fully recovered.” Nonetheless, based on this perceived recovery, he made the personal decision to decline further invasive follow-up investigations (e.g., CTA and endoscopy), while acknowledging the importance of monitoring. His experience profoundly altered his outlook on health: “I no longer take minor symptoms for granted.” He hopes that sharing his story encourages others to seek timely medical attention for seemingly trivial symptoms.

## Discussion

Our case, highlighting a life-threatening aortic injury, underscores a broader clinical phenomenon: migratory esophageal foreign bodies can precipitate a wide spectrum of vascular complications. Sharp objects may erode into other major vessels, including the carotid arteries, leading to pseudoaneurysm formation, catastrophic hemorrhage, or cerebrovascular events, emphasizing the need for high clinical vigilance regardless of the initial impaction site (2). Additionally, such migrations can cause mediastinal abscesses, pericardial effusions, and fistulous tracts to other structures like the trachea or pulmonary arteries. This broader context highlights that the clinical presentation and required intervention are dictated by the ultimate destination and involved anatomy of the migrating foreign body, not solely by its esophageal origin.

For patients presenting after accidental fish bone ingestion, contrast-enhanced CT is the preferred initial imaging modality for detecting retained bones, assessing extraluminal migration, and evaluating associated complications such as perforation or vascular injury (3). While endoscopy is a valuable tool for direct visualization and retrieval of intraluminal foreign bodies, its sensitivity for detecting complications like perforation or extraluminal migration is limited, with a non-negligible rate of false negatives (4). This is particularly true for sharp, thin objects like fish bones that may penetrate the wall rapidly and leave a minimal mucosal footprint. Therefore, persistent or suggestive symptoms (e.g., chest pain, fever, odynophagia) following an ingestion event, even if initial endoscopy is unremarkable or not performed, should always prompt urgent cross-sectional imaging (CT or CTA) (5). A high index of suspicion for extraluminal migration is crucial, even in cases where the patient does not recall a specific ingestion event.

Contrast-enhanced CT angiography (CTA) is the first-line imaging technique for diagnosing pseudoaneurysms (1). Thoracic endovascular aortic repair (TEVAR) is often the preferred initial treatment for rapid hemorrhage control (6). However, urgent open surgery was indicated in this case for several reasons:

1. **Anatomical Constraints:** The perforation site was located at the origin of the left common carotid artery (LCCA). TEVAR risked covering the LCCA and left subclavian artery (LSA) ostia and carried a significant risk of persistent endoleak-related hemorrhage from this specific location.
2. **Foreign Body Removal:** Open surgery was necessary to retrieve the migrated, sharp fish bone from the mediastinum.
3. **Infection Management:** The infected pseudoaneurysm required open debridement of inflammatory tissue and direct placement of postoperative irrigation drains.
4. **Surgical Strategy:** The planned approach—primary aortic perforation repair using autologous pericardium followed by the frozen elephant trunk (FET) procedure—minimized bleeding risk and isolated the infected cavity from the stent graft, reducing graft infection potential. The FET technique, standard for Stanford type A aortic dissection (7), is adaptable for aortic arch injuries from trauma or penetrating foreign bodies.Although deploying prosthetic material in a contaminated field is generally concerning, the FET technique in this context offers the advantage of excluding the perforation site and isolating the infected mediastinal cavity from the central aortic flow, which may mitigate the risk of graft infection when combined with radical debridement and prolonged postoperative irrigation (8).

Although secondary esophageal repair or reconstruction is commonly reported in the literature for such cases (9, 10), vigilant postoperative monitoring was implemented throughout the patient's recovery. This included tracking mediastinal drainage characteristics, body temperature patterns, and infectious biomarkers, with findings correlated against serial chest imaging. Convergent improvement across all monitored domains suggested a progressively increasing probability of esophageal fistula healing (11, 12). The patient's esophageal perforation healed completely within 6 weeks through postoperative gastrointestinal decompression and enteral nutrition support, obviating the need for reoperation.

Monitoring and managing the esophageal fistula constitute critical subsequent steps to determine whether conservative treatment achieves spontaneous closure or if secondary esophageal repair/reconstruction is required. Given the fistula presence, early mediastinal irrigation is imperative. Low negative pressure drainage serves the dual purpose of ensuring irrigation patency and preventing mediastinal fluid accumulation, which would impede sternal wound healing and risk cardiac tamponade (13). Antibiotic selection must cover polymicrobial infections involving oral flora, Gram-negative bacilli, and anaerobes. During surgery, a mediastinal irrigation tube and closed drainage system were placed at the pseudoaneurysm debridement site to facilitate enhanced postoperative monitoring and therapeutic mediastinal lavage (14). Endoscopic evaluation on postoperative day 2 revealed only a minor mucosal tear at the esophageal perforation site, supporting the decision for conservative management of the esophageal injury. Following 7 days of continuous mediastinal irrigation, the drainage fluid remained clear without evidence of infectious effusion. Concurrently, the patient maintained normal body temperature and exhibited unremarkable inflammatory biomarkers in blood tests, collectively suggesting probable healing of the esophageal perforation.

The strategic placement of a nasogastric tube for esophageal decompression proved critical in preventing digestive fluid leakage into the mediastinal cavity through the perforation site. Additionally, a jejunal feeding tube was employed to ensure adequate nutritional support throughout treatment. This integrated approach effectively balanced anatomical protection with metabolic demands during healing.

This case underscores that advanced imaging, meticulous surgical technique, and comprehensive postoperative care were critical to the successful resolution of this challenging clinical scenario. A notable limitation of this report is the relatively short follow-up period of 3 months and the patient's subsequent decline for recommended CTA and endoscopic reassessment. While the patient remains asymptomatic, this limits our ability to definitively confirm the long-term integrity of the aortic repair and complete esophageal healing. This limitation should be acknowledged when interpreting the outcomes. Extended clinical monitoring beyond this initial period is planned, including assessments of dietary status. The importance of compliance with follow-up imaging in such complex cases must be emphasized to patients despite an apparently successful initial recovery.

## Data Availability

The original contributions presented in the study are included in the article/Supplementary Material, further inquiries can be directed to the corresponding author.

## Supplementary material

Supplementary Video 1 The esophagography demonstrated that the esophageal wall has a continuous and smooth contour, with no filling defects, ulcerations, and no contrast extravasation into surrounding tissues.
